# Mapping trends and hotspots regarding the use of telenursing for elderly individuals with chronic diseases: A bibliometric analysis

**DOI:** 10.1097/MD.0000000000037313

**Published:** 2024-03-01

**Authors:** Yuan Yuan, Sican Wang, Chunhua Tao, Zhie Gu, Akio Kitayama, Kiyoko Yanagihara, Jingyan Liang

**Affiliations:** a School of Nursing and School of Public Health, Yangzhou University, Yangzhou, China; b Nagano College of Nursing, Komagane, Japan; c Subei People’s Hospital, Yangzhou, China; d Institute of Translational Medicine, Medical College, Yangzhou University, Yangzhou, China; e Jiangsu Key laboratory of integrated traditional Chinese and Western Medicine for prevention and treatment of Senile Diseases, Yangzhou University, Yangzhou, China

**Keywords:** bibliometric analysis, chronic diseases, CiteSpace, elderly, telenursing

## Abstract

**Background:**

Telenursing is receiving extensive attention from scholars and medical staff. However, there are few studies on the knowledge structure of telenursing for elderly individuals with chronic diseases. This study aims to demonstrate current research status and development trend of telenursing for elderly individuals with chronic diseases through a visual analysis of CiteSpace, so as to provide a more comprehensive perspective for future researches.

**Methods:**

Literature about telenursing for elderly patients with chronic diseases from 2002 to 2022 was retrieved from the Web of Science Core Collection using CiteSpace 6.1.R3.

**Results:**

A total of 375 records were obtained. Annual publication and citation frequency gradually increased over the investigated period, reaching a peak in 2022. Journal of Telemedicine and Telecare was the most prolific and the most cited journal. The United States was the most productive country, the University of Melbourne was the most productive institution, and the author CHEN C ranked the highest in the number of publications. The most popular keywords were “care,” “telemedicine,” “management,” “older adult,” “chronic disease,” “health,” and “heart failure,” which had a high frequency and centrality. The keywords “telehealth,” “randomized controlled trail,” “chronic obstructive pulmonary disease,” “implementation” and “time” showed the strongest citation burst. The keywords were clustered to form 10 labels. The article published in 2010 by Chaudhry SI was cited the most. The top 3 cited journals were all special journal of telemedicine.

**Conclusion:**

This study revealed current research status and development trend of telenursing for elderly individuals with chronic diseases. The bibliometric analysis of telenursing expands the knowledge field of telemedicine and provides new insights into the management of elderly patients with chronic diseases.

## 1. Introduction

Population aging is one of the most important global trends in the 21st century.^[1]^ The World Report on Ageing and Health published by the World Health Organization, confirms that at present, the life expectancy of most people in the world can exceed 60 years currently.^[2]^ With the aggravation of population aging, the main burden of diseases has shifted to chronic non-communicable diseases. The death caused by chronic diseases has become a major public health problem, which not only affects the health of residents but also affects the economic and social development of the country.^[3]^ Therefore, it is of great significance to achieve effective management of elderly individuals with chronic diseases.^[4]^

In recent years, with the development of information technology, the advantages of telemedicine have become increasingly prominent.^[5]^ Telemedicine can effectively overcome the obstacles of time and space, improve the efficiency and effect of diagnosis and treatment, reduce medical costs, and improve the quality of chronic disease management. Information technology also shows an incredible prospect in the field of nursing, which promotes the birth of a new branch of telemedicine, namely telenursing.^[6]^ There are slight differences in the definition of telenursing among different literature, overall, telenursing refers to “delivery of nursing services via remote telecommunications.”^[7]^ Telenursing is receiving extensive attention from scholars and medical staff. Researches has shown that compared with traditional face-to-face nursing, telenursing is equally effective, or even more effective, with lower cost, for example, for the elderly individuals with chronic diseases.^[8]^ Especially during the pandemic of Corona Virus Disease 2019 (COVID-19), elderly individuals with chronic diseases are at high risk of neocoronal pneumonia.^[9]^ Prevention and control measures such as reduction of population movement can effectively prevent the elderly from infecting neocoronal pneumonia. However, the elderly need to leave home for regular checkups and purchasing medication, receiving treatment and nursing, representing a major conflict with epidemic prevention policies. In this case, telenursing seems to be a viable alternative to face-to-face nursing services.^[10]^ Therefore, nowadays, telenursing has been paid more and more attention, especially for the elderly individuals with chronic diseases. However, there are few studies on the comprehensive knowledge structure of telenursing for elderly individuals with chronic diseases.

Bibliometrics can visualize the potential knowledge contained in scientific documents, and can be used to explore research hotspots, research frontiers, and predict the future development trend of a certain research field.^[11]^ Lan et al^[12]^ used bibliometrics to explore application of telemedicine in COVID-19 on the retrieved 5224 papers from PubMed. Waqas et al^[13]^ presented a comprehensive knowledge structure of telemedicine from Web of Science in 2010 to 2019 based on CiteSpace. However, there is no visual research on the comprehensive knowledge structure of telenursing for elderly individuals with chronic diseases from the perspective of bibliometrics. Since the outbreak of COVID-19, the demand for telenursing has been increasing, more and more researchers have participated in the study of telenursing.^[14]^ Bibliometrics research on telenursing can provide new enlightenment for nursing, and to promote the further development of telemedicine.

CiteSpace is a commonly used bibliometric analysis tool, which can intuitively provide researchers with knowledge structure information and potential research directions.^[15]^ Therefore, the purpose of this study is to use CiteSpace to analyze the records retrieved from Web of Science Core Collection (WOSCC) in the past 20 years, so as to better understand the current research status and development trend of telenursing for elderly individuals with chronic diseases, and then guide future researches for the researchers in this field.

## 2. Materials and methods

### 2.1. Data collection

**Figure 1.**
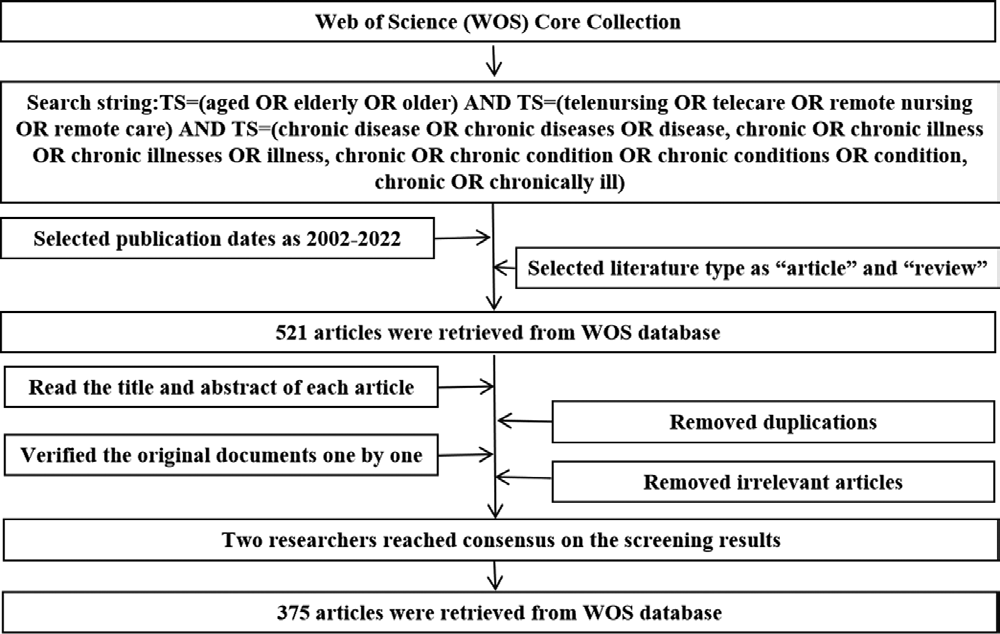
Data collection process.

The data of this study were collected from the WOSCC including Science Citation Index Expanded, Social Sciences Citation Index, Arts and Humanities Citation Index, Emerging Sources Citation Index, Current Chemical Reactions, and Index Chemicus in 2022. Topic (TS) was used for data search, the data search string was as follows: TS = (aged OR elderly OR older) and TS = (telenursing OR telecare OR remote nursing OR remote care) and TS = (chronic disease OR chronic diseases OR disease, chronic OR chronic illness OR chronic illnesses OR illness, chronic OR chronic condition OR chronic conditions OR condition, chronic OR chronically ill). The retrieval period time was from January 2002 to December 2022. Languages were no restrictions and 524 records were retrieved. The literature types as “article” and “review” were retained, 1 editorial material, 1 letter, and 1 meeting abstract were removed, remaining 521 records. By reading the titles and abstracts of the articles, the duplicate articles were manually deleted. By verifying the original documents one by one, we removed the irrelevant articles, those whose research subjects were not the elderly, those whose research diseases were not chronic diseases, and those that did not use telenursing. Finally, 2 researchers reached consensus on the screening results and 375 articles were included. Figure 1 shows the data collection process.

Because all data were obtained from a publicly available database, this study does not require ethical approval.

### 2.2. Data analysis tool

CiteSpace is a document analysis software based on Java, developed by Professor Chaomei Chen from Drexel University in America.^[16]^ It can visualize the relationship between scientific papers according to their co-citation patterns. Visual knowledge maps usually show networks as types of node-and-link diagrams. Nodes can represent keyword, country, institution, author and so on. The size of nodes usually indicates the frequency of occurrence or being referenced, and different colors of nodes show different years. Links between nodes represent co-occurrence or collaboration or co-citation. Nodes with centrality >0.1 are usually considered as key points or turning points in specific fields. Additionally, CiteSpace provides module value (Q value) and average contour value (S value) based on network structure and clustering clarity, these 2 indicators are used as the basis for judging the mapping effect. In general, Q > 0.3 indicates community structure is significant, and S > 0.5 indicates clustering is reasonable.^[17]^ In this study, the version of CiteSpace was 6.1.R3 (64-bit), Time Slicing was “2002-2022,” Years Per Slice was “1,” Term Source was “all selection,” Node types was “choose one at a time,” Selection Criteria was “top 50 levels,” pruning was “pruning sliced networks,” and visualization was “cluster view-static.”

## 3. Results and discussion

### 3.1. Literature distribution of journal papers related to telenursing for elderly individuals with chronic diseases

#### 3.1.1. Annual publication and citation frequency of telenursing for elderly individuals with chronic diseases.

**Figure 2.**
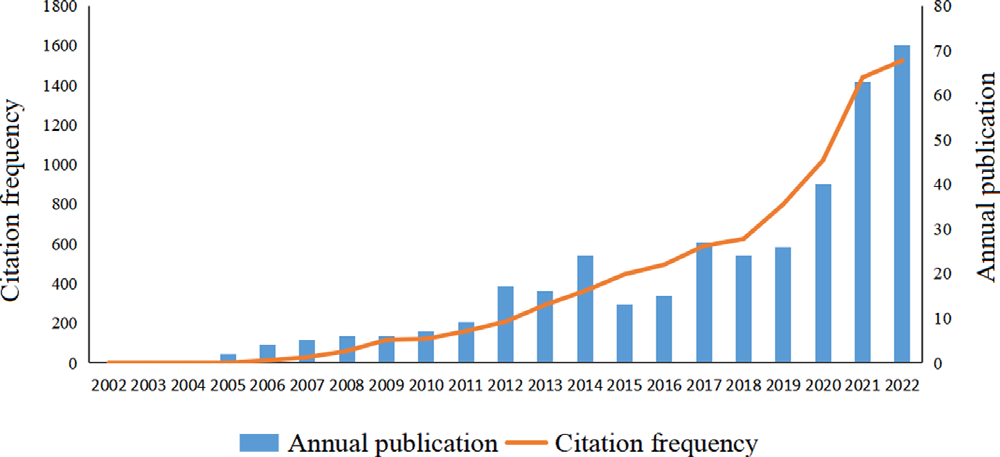
Annual publication and citation frequency of telenursing for elderly individuals with chronic diseases in recent 20 years.

The changes of publication numbers and citation frequencies can intuitively reflect the changes of research hotspots in a certain research field in a specific period of time.^[18]^ In the past 20 years, the literature related to telenursing for elderly individuals with chronic diseases has shown an upward trend. In 2022, the number reached the peak, with 71 posts. The citation frequency of relevant literature on telenursing for elderly individuals with chronic diseases has also steadily increased, reaching a peak in 2022, with a citation frequency of 1524, which may be due to the COVID-19 pandemic, which makes people pay more attention to telenursing. The number of publication and citation frequency are shown in Figure 2. These results indicated that telenursing, as a medical technology bred in a new era, is receiving more and more attention and more relevant researches are being carried out.^[19]^

#### 3.1.2. The most productive journals related to telenursing for elderly individuals with chronic diseases.

The top ten productive journals related to telenursing for elderly individuals with chronic diseases are listed in Table 1. Of the ten journals, the average impact factor is 4.537. The top 3 journals are Journal of Telemedicine and Telecare, Telemedicine and e-Health, Journal of Medical Internet Research, and the impact factors of the top 3 are all exceeded 5. This fully proved that at present, the attention of telenursing is very high.

**Table 1:** The most productive journals related to telenursing for elderly individuals with chronic diseases.

Rank	Journals	Publications	IF
1	Journal of Telemedicine and Telecare	21	6.344
2	Telemedicine and e-Health	20	5.033
3	Journal of Medical Internet Research	17	7.076
4	International Journal of Medical Informatics	8	4.730
5	Rural and Remote Health	8	2.733
6	International Journal of Environmental Research and Public Health	7	4.614
7	JMIR Medical Informatics	6	3.228
8	BMC Health Services Research	5	2.908
9	JMIR mHealth and uHealth	5	4.947
10	PLoS One	5	3.752

IF = impact factor, referred to Journal Citation Reports (2022).

#### 3.1.3. The most productive countries related to telenursing for elderly individuals with chronic diseases.

**Figure 3.**
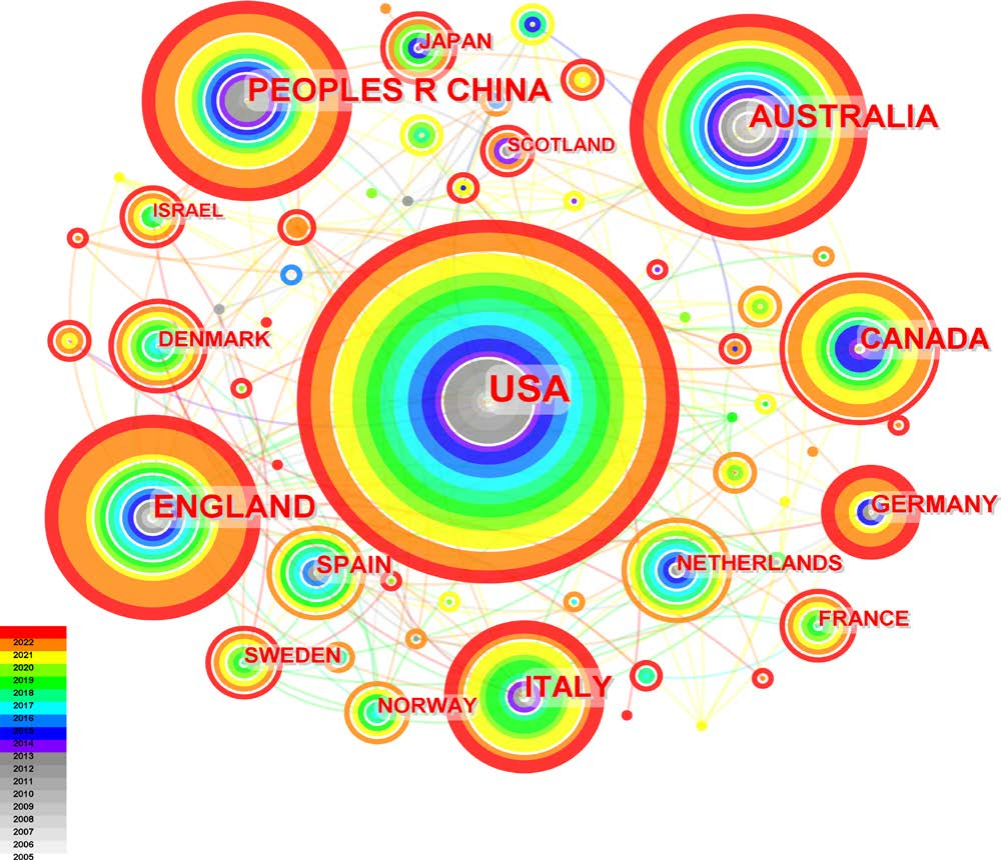
Map of most productive countries related to telenursing for elderly individuals with chronic diseases in recent 20 years.

Telenursing has attracted attention in many countries.^[20]^ Table 2 and Figure 3 show the the most productive countries related to telenursing for elderly individuals with chronic diseases based on CiteSpace. The included 375 articles were published by researchers in 55 countries. The main contributor was USA, accounting for about a fourth of the total articles (89). England and Australia ranked second and third, respectively. In addition, almost all the most productive countries are developed countries, which may be due to the fact that higher economy and technology are conducive to the development of telenursing.^[21]^ In Figure 3, the nodes represented the countries, the colors indicates the years of publication, the closer it is to red, the newer it is, and the closer it is to light gray, the older it is. In addition, the larger the circle, the larger the number of populations. And as can be seen from the figure, there has been a lot of cooperation between different countries, especially in the past 5 years.

**Table 2:** The most productive countries related to telenursing for elderly individuals with chronic diseases.

Number	Rank	Publications	Countries	Year of first publication
1	1	89	USA	2006
2	2	44	England	2006
3	3	43	Australia	2005
4	4	35	People’s Republic of China	2009
5	5	28	Italy	2005
6	6	26	Canada	2007
7	7	14	Spain	2008
8	8	13	Germany	2009
9	9	11	France	2016
10	9	11	Denmark	2011

#### 3.1.4. The most productive institutions related to telenursing for elderly individuals with chronic diseases.

**Figure 4.**
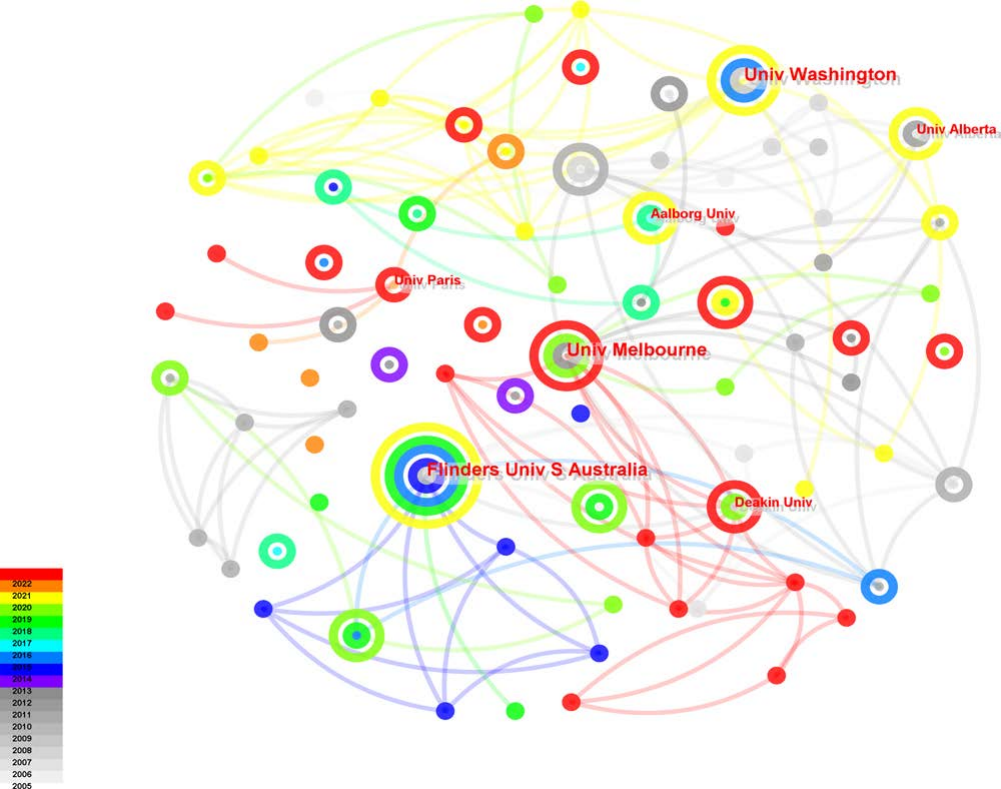
Map of most productive institutions related to telenursing for elderly individuals with chronic diseases in recent 20 years.

Of the 364 institutions which focused on telenursing for elderly individuals with chronic diseases (Figure 4; Table 3), the top 8 institutions were all universities and the top 3 institutions were University of Melbourne, Flinders University, University of Washington. They were the cores of forming a complex cooperation network, especially University of Melbourne. University of Melbourne research on telenursing for elderly individuals with chronic diseases involved chronic obstructive pulmonary disease (COPD), Chronic kidney disease, diabetes, and so on. In addition, University of Melbourne not only did observational studies, but also explored the role of telenursing during the COVID-19 pandemic.

**Table 3:** The most productive institutions related to telenursing for elderly individuals with chronic diseases.

Number	Rank	Publications	Institutions	Year of first publication
1	1	7	University of Melbourne	2006
2	2	6	Flinders University	2006
3	2	6	University of Washington	2005
4	4	4	University of Paris	2009
5	4	4	Deakin University	2005
6	4	4	University of Sheffield	2007
7	4	4	University of Alberta	2008
8	4	4	Aalborg University	2009

#### 3.1.5. The most productive authors related to telenursing for elderly individuals with chronic diseases.

**Figure 5.**
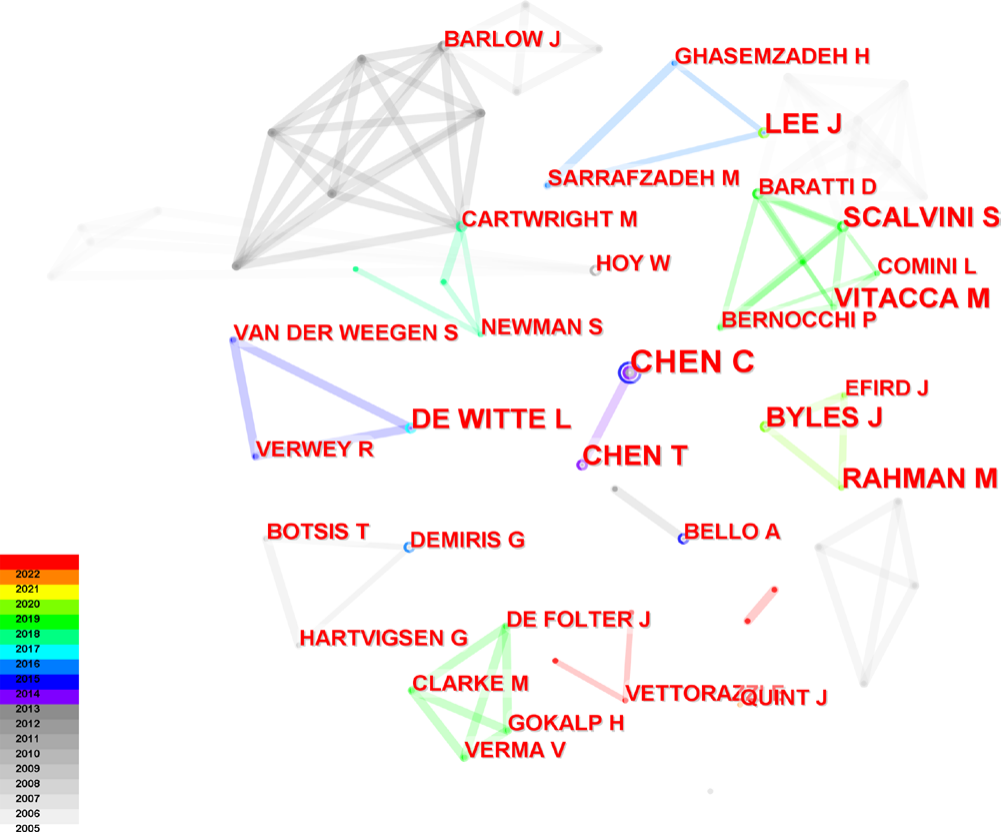
Map of most productive authors related to telenursing for elderly individuals with chronic diseases in recent 20 years.

The authors of the 375 publications were analyzed, 446 nodes and 662 links were obtained, as shown in Figure 5 and Table 4, indicating that 375 articles were published by 446 authors. Among these most productive authors, there were both interconnected collaborative groups and decentralized individual authors. RAHMAN M of Hiroshima University in Japan had the most articles in the past 3 years. He has conducted researches on telenursing in both Japan and China. He found telenursing might effectively improve the lifestyles-related behaviors of patients with chronic illness on the remote island.^[22]^ In addition, RAHMAN M found nurse-led collaborative management using remote monitoring might improve the psychosocial status of patients with heart failure and prevent rehospitalization due to heart failure.^[23]^ Additionally, he found telephone counseling can help patients gain self-management skills to cope with their illness and significantly improve their quality of life.^[24]^

**Table 4:** The most productive authors related to telenursing for elderly individuals with chronic diseases.

Number	Rank	Publications	Authors	Year of first publication
1	1	4	CHEN C	2009
2	2	3	RAHMAN M	2019
3	2	3	VITACCA M	2018
4	2	3	LEE J	2015
5	2	3	DE WITTE L	2014
6	2	3	CHEN T	2012
7	2	3	BYLES J	2010
8	2	3	SCALVINI S	2005

### 3.2. Hotspots and frontiers analysis of journal papers related to telenursing for elderly individuals with chronic diseases

#### 3.2.1. The most popular keywords related to telenursing for elderly individuals with chronic diseases.

**Figure 6.**
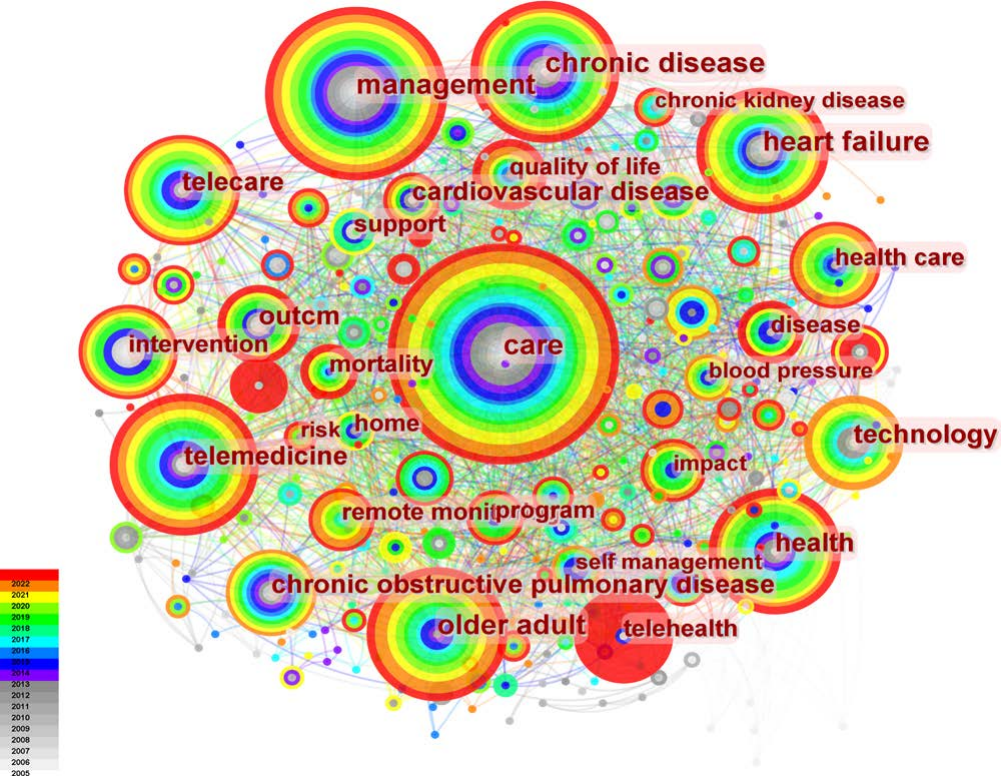
Map of most popular keywords related to telenursing for elderly individuals with chronic diseases in recent 20 years.

It was believed that the indexes of evaluating the most cutting-edge topics or emerging trends were the increase of keyword frequency or the increased number of keyword bursts in the citation within a certain period of time.^[25]^ A keywords network map was generated, consisting of 380 nodes and 2087 links, as seen in Figure 6. This study identified 380 research keywords in the field of telenursing for the elderly individuals with chronic diseases, revealing the most popular topics at present. According to the centrality and frequency in Table 5, we can see that the most popular keywords were “care,” “telemedicine,” “management,” “older adult,” “chronic disease,” “health,” and “heart failure,” which all had a high centrality and frequency. Among these words, we found, at present, the most concerned patients of telenursing are heart failure patients. Population aging has led to an increase in the incidence of multiple diseases. Globally, 3 quarters of the elderly individuals suffer from multimorbidities with cardiovascular disease (CVD), accounting for 31% of the world’s deaths. 64.3 million people suffer from heart failure, which accounts for about one third of CVDs.^[26]^ Researches found that in primary health care and community settings, integrated telenusing can effectively reduce the mortality of hospitalized, rehospitalized, and elderly morbid adults related to CVDs. However, due to differences in telehealth modalities and the risk of bias, there are many differences between studies.^[27]^ Furthermore, we can also find some words like “quality of life” in the figure. The frequency of the keyword “quality of life” has been increasing year by year, indicating that people are very concerned about the quality of life of elderly individuals with chronic diseases after telenursing, both the physical and mental changes.^[28]^ The main measurements included EQ-5D-3L, SF-36 questionnaires.^[29,30]^

**Table 5:** The top 10 frequency and centrality of keywords related to telenursing for elderly individuals with chronic diseases.

Rank	Keyword	Frequency	Rank	Keyword	Centrality
1	care	79	1	chronic disease	0.26
2	telemedicine	53	2	care	0.17
3	management	45	3	management	0.16
4	older adult	43	4	older adult	0.14
5	chronic disease	41	5	heart failure	0.13
6	health	36	6	telemedicine	0.12
7	heart failure	34	7	telecare	0.12
8	telehealth	30	8	health	0.10
9	telecare	29	9	cardiovascular disease	0.10
10	technology	28	10	technology	0.07

**Figure 7.**
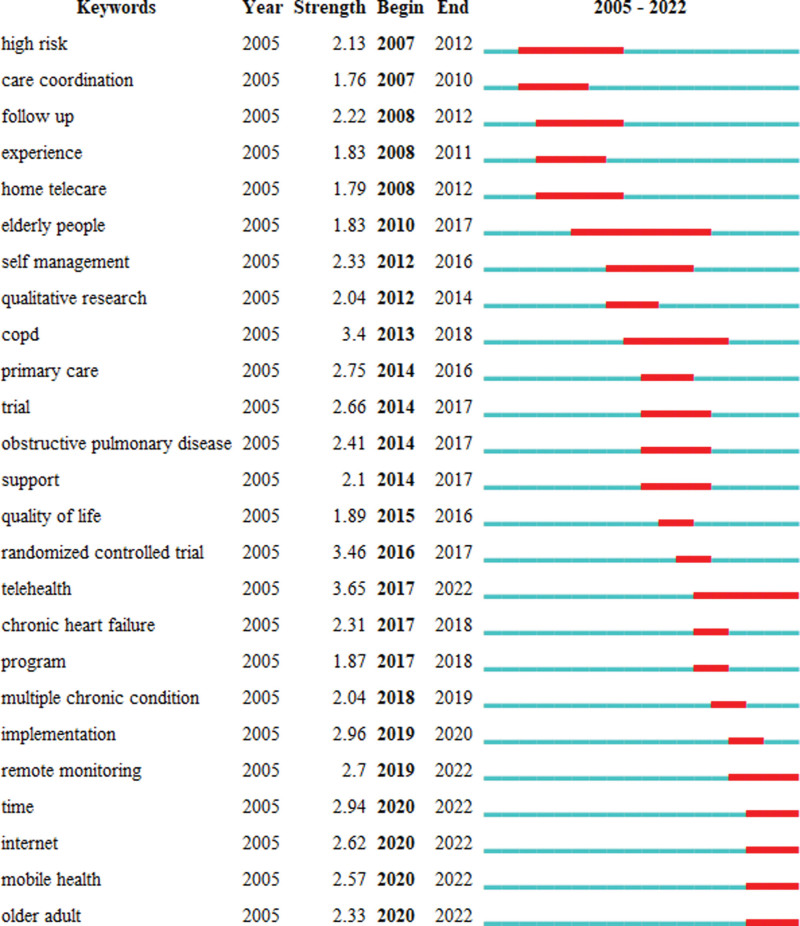
Top 25 keywords with the strongest citation bursts.

Figure 7 shows the top 25 cited keywords with the strongest citation bursts in 20 recent years, and the top 5 is “telehealth,” “randomized controlled trail,” “COPD,” “implementation” and “time.” The first cited keyword with the strongest citation burst is “telehealth.” Telehealth is, in fact, telemedicine, which means “Delivery of health services via remote telecommunications. This includes interactive consultative and diagnostic services.”^[31]^ While telenursing means “Delivery of nursing services via remote telecommunications.”^[32]^ So telenursing is, in a sense, a branch of telehealth. The second one is “randomized controlled trail.” A randomized controlled trial is a gold standard to evaluate the efficacy of telenursing, it is the best way to provide high-quality evidence-based testimony.^[33]^ The third one is “COPD,” according to the World Health Organization (WHO), COPD has become the third leading cause of death, and age is the main cause of major events. For patients with COPD, nurses enhanced patient care by using telecommunications technology, they used the electromagnetic channels to transmit data, voice, and video.^[34]^ The fourth one is “implementation.” After putting forward a concept, such as telenursing, the most important thing is the implementation, and then analyze the effects of the implementation, summarize the importance of the implementation, the problems encountered in the implementation process, and the challenges of future implementation. Only in this way can we continuously promote the optimization of telenursing and promote the health of patients. In recent years, researches on telenursing has done this very well. The fifth one is “time.” Many studies have proposed that telenursing can monitor the relevant data of patients in real time, and patients can consult nurses at any time.^[35]^ Additionally, telenursing saved patients’ time and reduced hospitalization time. The most recent burst keywords are “remote monitoring,” “time,” “internet,” “mobile health,” “older adult,” this are the research hotspots and frontiers in recent years. Among the 5 key words, what deserves our attention most is “remote monitoring.” With the improvement of science and technology and the development of economy, more and more remote monitoring projects are with higher accuracy. More and more countries begin to use remote monitoring, and more and more families have the financial ability to afford remote monitoring.^[36]^ But the effects of different remote monitoring systems are not very clear, so more and more researchers are analyzing the effects of various remote monitoring systems, for example, Liu et al^[37]^ revealed the application prospects of deep learning and artificial intelligence in skin flap monitoring.

#### 3.2.2. Clusters analysis of keywords related to telenursing for elderly individuals with chronic diseases.

**Figure 8.**
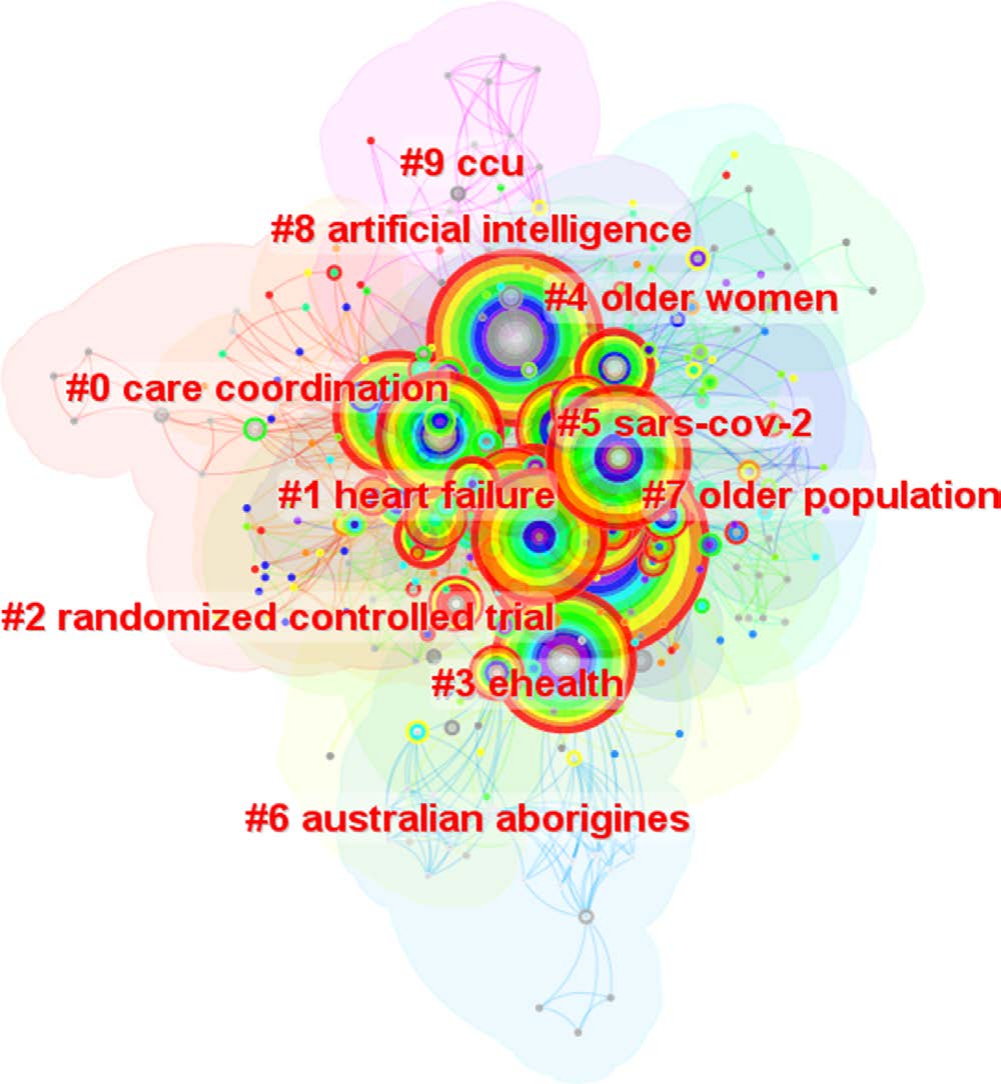
Map of keywords clusters related to telenursing for elderly individuals with chronic diseases in recent 20 years (Q = 0.4071, S = 0.7659).

We carried out cluster analysis on keywords and summarized them, so as to more intuitively understand the current research topics related to telenursing for elderly individuals with chronic diseases. After clustering, Q value is 0.4071 and S value is 0.7659, which indicates that this clustering is appropriate and meaningful. 10 clusters have been generated to reflect the hot trends, among them, the top 6 clusters which contain the most keywords are “care coordination,” “heart failure,” “randomized controlled trail,” “ehealth,” “older women” and “sars-cov-2,” as shown in Figure 8 and Table 6. The first cluster is care coordination. Care coordination is a healthcare approach in which all patients’ needs are coordinated with the assistance of a primary point of contact.^[38]^ Researches found the effects of disease management using telemonitoring for patients remained controversial. However, when care coordination and enhanced collaborative self-management were embedded, it was more conducive to the improvement of the patient’s health.^[39]^ It illustrated the importance of nurses during telemedicine, which was what we call telenursing. The second cluster is “heart failure,” as mentioned earlier, due to the high prevalence of heart failure in the elderly, telenursing alleviated a great medical burden. The third clusters is “randomized controlled trail,” as previously mentioned, a randomized controlled trial provides high-quality evidence-based testimony. The fourth cluster is “ehealth,” as mentioned earlier, e-health is, in fact, telemedicine, while telenursing is, in a sense, a branch of e-Health. The fifth cluster is “older women.” Some researches found older women were more likely than men to use telenursing, especially during the pandemic. For fear of getting infected, older women would take many measures, such as maintaining social distancing.^[40]^ Telenursing can reduce the times patients go to the hospital, which is a good way to maintain social distance. The sixth clusters is “sars-cov-2.” Sars-cov-2 is a species corona virus causing COVID-19 in humans. The outbreak of COVID-19 further accelerated the deployment and utilization of telenursing services.^[41]^

**Table 6:** Top 10 keywords clusters related to telenursing for elderly individuals with chronic diseases.

Number	Cluster ID	Clustering tags	Size	Silhouette	Mean (year)
1	0	Care coordination	41	0.739	2012
2	1	Heart failure	41	0.809	2016
3	2	Randomized controlled trail	40	0.754	2013
4	3	e-Health	38	0.701	2014
5	4	Older women	37	0.712	2012
6	5	SARS-CoV-2	37	0.713	2015
7	6	Australian aborigines	36	0.877	2008
8	7	Older population	31	0.769	2014
9	8	Artificial intelligence	31	0.72	2013
10	9	CCU	21	0.89	2011

**Figure 9.**
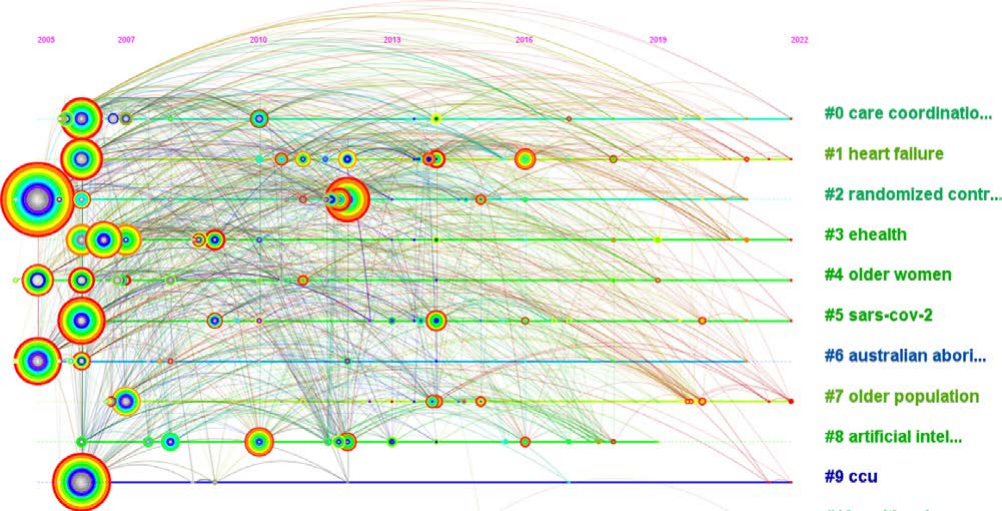
Timeline of keywords clusters related to telenursing for elderly individuals with chronic diseases in recent 20 years.

Figure 9 shows the timeline of keywords clusters related to telenursing for elderly individuals with chronic diseases from 2002 to 2022. In fact, almost all the keywords clusters appeared early because the keywords included in these clusters may appear earlier. For example, sars-cov-2, although it was only been around since late 2019, the cluster includes keywords like “anxiety” and “depression.” Before 2019, researchers confirmed that telenursing could effectively improve anxiety and depression symptoms in elderly individuals with chronic diseases.^[42]^ The more red lines clustered in Figure 9, the more they have been mentioned in recent years, indicating that “older population” has attracted more and more attention from researchers, because it is indeed a global problem and should attract the attention of medical decision makers.^[43]^

#### 3.2.3. Analysis of cited references related to telenursing for elderly individuals with chronic diseases.

**Figure 10.**
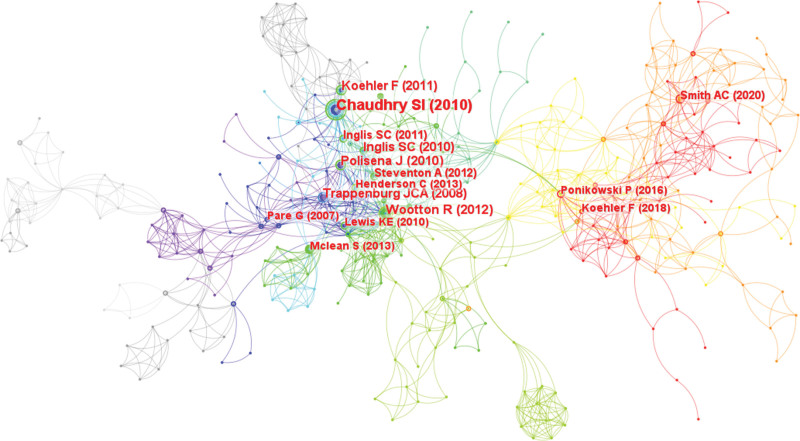
Map of cited references related to telenursing for elderly individuals with chronic diseases in recent 20 years.

The results of a cited reference co-citation map includes 601 nodes and 1796 links, as shown in Figure 10. Table 7 shows the most popular cited references, the first is the paper published in 2010 by Chaudhry SI.^[44]^ In fact, the article found telemonitoring did not improve the results of patients recently hospitalized for heart failure. The article presented the importance of a thorough and independent assessment of disease management strategies before the adoption. Based on the suggestions, many researchers rectified the implementation of telenursing and achieved better results. Another article worthy of our attention was published by Smith AC in 2020.^[50]^ Although it has only been published for 2 years, its citation times have reached top 7. The article believed that the current COVID-19 pandemic reminded us again of the importance of using telehealth to provide care, especially as means to reduce the risk of cross-contamination caused from close contact.

**Table 7:** The most popular cited references related to telenursing for elderly individuals with chronic diseases.

Rank	Title	Author	Year	Journal	IF
1	Telemonitoring in patients with heart failure	Chaudhry et al^[44]^	2010	The New England Journal of Medicine	176.079
2	Twenty years of telemedicine in chronic disease management--an evidence synthesis	Wootton R^[45]^	2012	Journal of Telemedicine and Telecare	6.344
3	Home telehealth for chronic obstructive pulmonary disease: a systematic review and meta-analysis	Polisena et al^[46]^	2010	Journal of Telemedicine and Telecare	6.344
4	Impact of remote telemedical management on mortality and hospitalizations in ambulatory patients with chronic heart failure	Koehler et al^[47]^	2011	Circulation	39.918
5	Effects of telemonitoring in patients with chronic obstructive pulmonary disease	Trappenburg et al^[48]^	2008	Telemedicine and-e-Health	5.033
6	Structured telephone support or telemonitoring programmes for patients with chronic heart failure	Inglis et al^[49]^	2010	The Cochrane Database of Systematic Reviews	12.008
7	Telehealth for global emergencies: Implications for coronavirus disease 2019 (COVID-19)	Smith et al^[50]^	2020	Journal of Telemedicine and Telecare	6.344
8	Effect of telehealth on use of secondary care and mortality: findings from the Whole System Demonstrator cluster randomized trial	Steventon et al^[51]^	2012	BMJ-British Medical Journal	93.333
9	Which components of heart failure programmes are effective?	Inglis et al^[52]^	2011	European Journal of Heart Failure	17.349

IF = impact factor, referred to Journal Citation Reports (2022).

**Figure 11.**
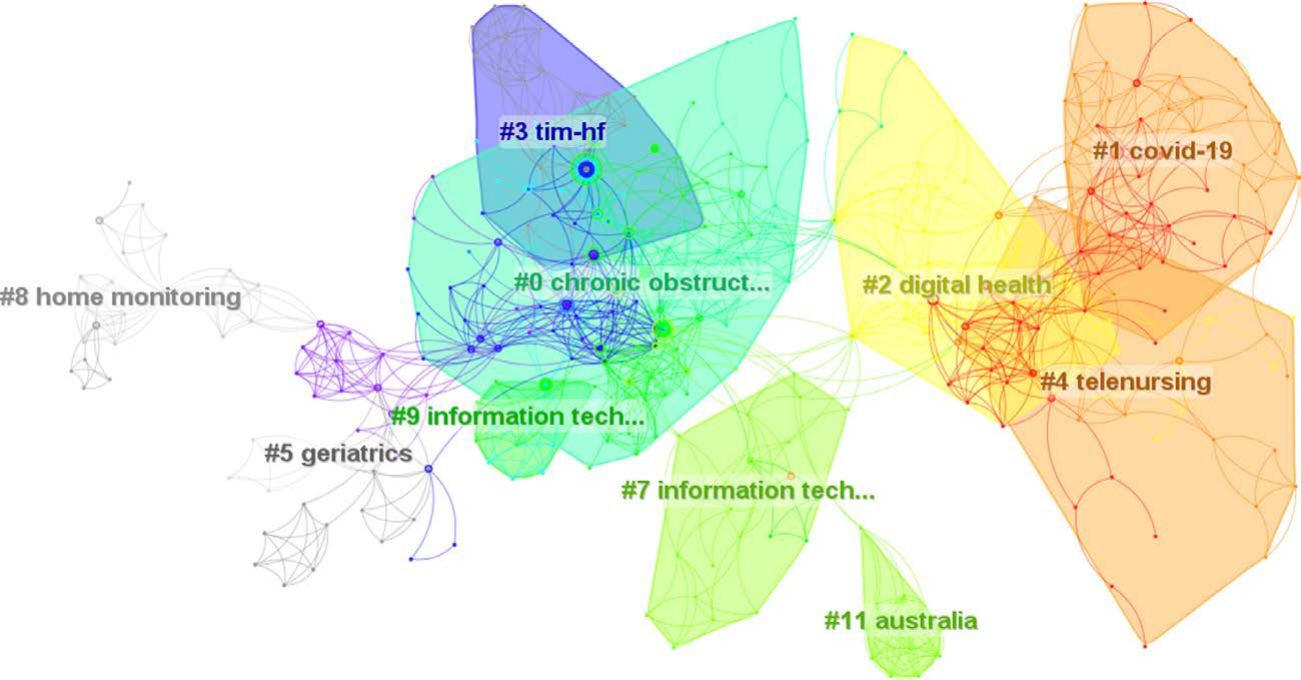
Map of cluster analysis on the references related to telenursing for elderly individuals with chronic diseases in recent 20 years (Q = 0.8512, S = 0.9361).

**Figure 12.**
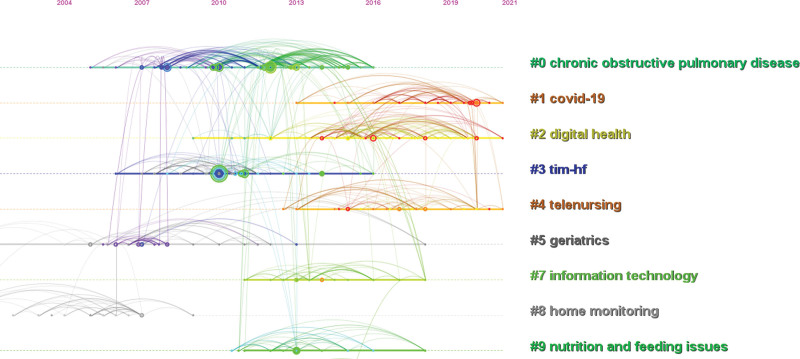
Timeline of cluster analysis on the references related to telenursing for elderly individuals with chronic diseases in recent 20 years.

We carried out cluster analysis and timeline on the references and then summarized them (Figures 11 and 12), the top 3 are “chronic obstructive pulmonary disease,” “covid-19” and “digital health.” This shows that many studies were focusing on telenursing for COPD, and more and more studies were focusing on the importance of telenursing using digital devices during the COVID-19 pandemic in recent years. Xie et al^[53]^ proposed that some digital devices were used in the field of health, they had the advantages of being low-cost, continuously monitorable, noninvasive, remotely observable, and with automatic alarms. However, differences in digital health are a common challenge,^[54]^ researchers proposed that in the future, large-scale studies are needed to find more accurate and reliable values in the corresponding field, further improving the accuracy of digital health.^[55]^

#### 3.2.4. Analysis of cited journals related to telenursing for elderly individuals with chronic diseases.

**Figure 13.**
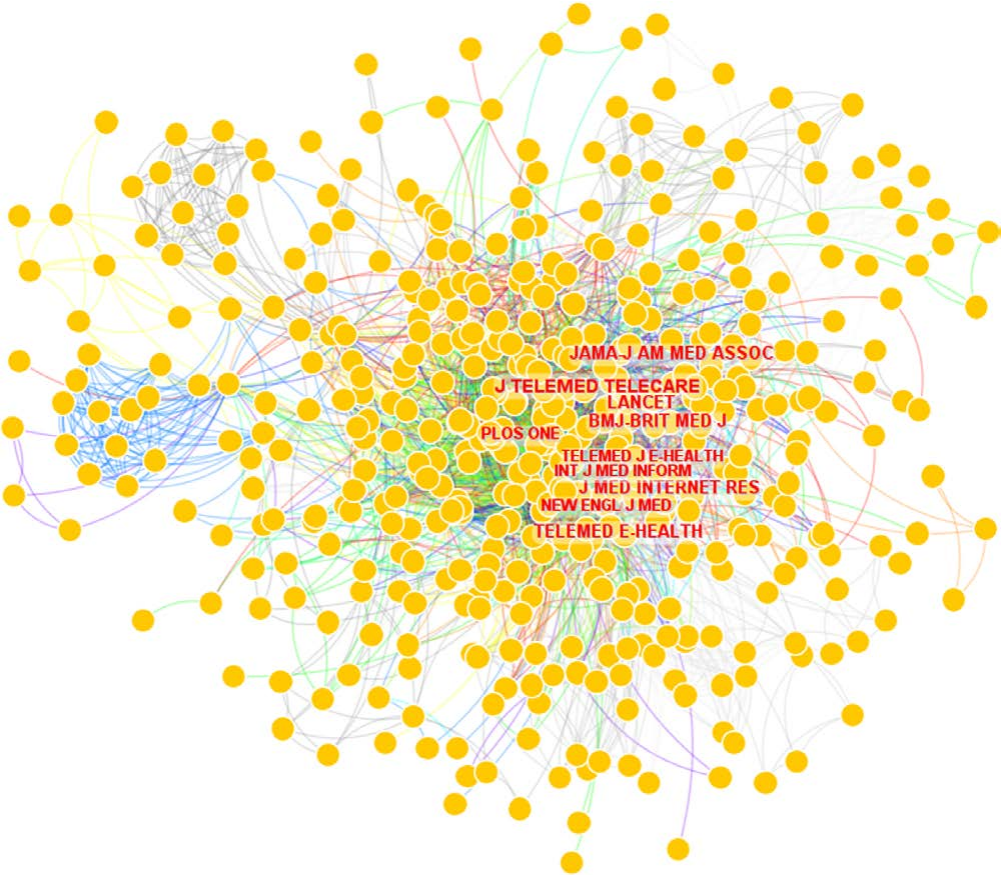
Map of cited journals related to telenursing for elderly individuals with chronic diseases in recent 20 years.

As shown in Figure 13, CiteSpace generated a map of cited journals, including 490 nodes and 3222 links. In the map, nodes represented journals, links between the nodes represented co-citation relationships. The top 10 cited journals related to telenursing for elderly individuals with chronic diseases are shown in Table 8. Among them, the top 3 cited journals are all special journal of telemedicine. These are journals that we can refer to when we implement telenursing research in the future. The average impact factor is 46.952 and the top 3 journal of highest impact factors are “Lancet,” “New England Journal of Medicine” and “BMJ-British Medical Journal.” The citation of a single article in these top journals is often particularly high, so the articles on telenursing published in these journals are often worth our in-depth study.

**Table 8:** Top 10 cited journals related to telenursing for elderly individuals with chronic diseases.

Number	Rank	Frequency	Journals	IF
1	1	132	Journal of Telemedicine and Telecare	6.344
2	2	115	Journal of Medical Internet Research	7.076
3	3	97	Telemedicine and e-Health	5.033
4	4	78	PLoS One	3.752
5	5	76	Lancet	202.731
6	6	70	BMJ-British Medical Journal	93.333
7	7	67	International Journal of Medical Informatics	4.730
8	8	61	Cochrane Database of Systematic Reviews	12.008
9	9	56	New England Journal of Medicine	176.079
10	9	56	BMC Health Service Research	2.908

IF = impact factor, referred to Journal Citation Reports (2022).

## 4. Conclusion

As an important part of telemedicine, telenursing supported by telecommunication technology has gradually become the focus of researchers, especially for the elderly individuals with chronic diseases. Nowadays, the researches about telenursing for elderly individuals with chronic diseases mainly carried out in developed countries, and the authors have formed some cooperative networks, but the global cooperation on telenursing can be further strengthened. Current randomized controlled trials and reviews have demonstrated that the intervention effects of telenursing are equivalent to traditional face-to-face nursing but more cost-effective. For now, the main focus area of telenursing are COPD and heart failure, and in the future, researches may turn to the applicability of real time remote monitoring using electronic devices in the era of pandemic. At the same time, telenursing may pay more attention to care coordination and may develop personalized nursing plans. This will not only satisfy patients, but also gets the approval of the family and nurses. In the future, more researches about telenursing with the support of emerging technologies can be conducted to further improve the health condition and quality of life of elderly individuals with chronic diseases, leading to a longer, healthier, and happier life.

### 4.1. Limitations

There are some limitations in this study. First, although WOSCC is widely recognized, some literatures have also been retained on other data platforms. The reason is that some databases are limited in the use of CiteSpace, which can be resolved in future updates to related analysis software. Second, only relevant information of journals, countries, institutions, authors, keywords and citations were analyzed, but each article contains rich information, further in-depth research can be conducted in the future to obtain more detailed and comprehensive knowledge map.

## Acknowledgments

The authors would like to thank librarians from Yangzhou University, for their suggestions and assistance in data acquisition and bibliometric analysis. The authors also would like to thank the funders, because this work was supported by National Natural Science Foundation of China (82300466), the General Program of Natural Science Research in Colleges and Universities of Jiangsu Province (21KJD320005), and the Open Project of Key Laboratory of Animal Genetic Breeding and Molecular Design in Jiangsu Province (AGBMD2021).

## Author contributions

**Conceptualization:** Yuan Yuan, Sican Wang, Chunhua Tao, Jingyan Liang.

**Data curation:** Yuan Yuan, Sican Wang, Chunhua Tao, Jingyan Liang.

**Formal analysis:** Yuan Yuan, Zhie Gu, Jingyan Liang.

**Funding acquisition:** Yuan Yuan, Zhie Gu, Jingyan Liang.

**Investigation:** Yuan Yuan, Sican Wang, Chunhua Tao, Jingyan Liang.

**Methodology:** Yuan Yuan, Sican Wang, Chunhua Tao, Jingyan Liang.

**Project administration:** Yuan Yuan, Sican Wang, Chunhua Tao, Jingyan Liang.

**Resources:** Yuan Yuan, Akio Kitayama, Kiyoko Yanagihara, Jingyan Liang.

**Software:** Yuan Yuan, Akio Kitayama, Kiyoko Yanagihara, Jingyan Liang.

**Supervision:** Yuan Yuan, Akio Kitayama, Kiyoko Yanagihara, Jingyan Liang.

**Validation:** Yuan Yuan, Akio Kitayama, Kiyoko Yanagihara, Jingyan Liang.

**Visualization:** Yuan Yuan, Akio Kitayama, Kiyoko Yanagihara, Jingyan Liang.

**Writing—original draft:** Yuan Yuan, Chunhua Tao.

**Writing—review & editing:** Jingyan Liang.
